# Supplementary material for: Association of Hours of Paid Work with Dietary Intake and Quality in Japanese Married Women: A Cross-Sectional Study
Source: Nutrients. 2021 Aug 28;13(9):3005. doi: 10.3390/nu13093005 (PMC8466932; doi:10.3390/nu13093005)
Supplement: Supplementary file 1 [file nutrients-13-03005-s001.zip › nutrients-1274960-supplementary.pdf]

Table S1. Reference daily values used for the calculation of the NRF9.3 for Japanese women aged 20-59 years[a] and component and total scores of NRF9.3 in 644 Japanese married women.

	Reference daily values			NRF9.3 score		n (%) of participants meeting RDVs
	20–29 years	30–49 years	50–59 years	Mean	SD	
Total NRF9.3 score	-	-	-	682	102	-
Energy (kcal)[b]	1950	2000	1900	-	-	-
Qualifying nutrients						-
Protein (g)[c]	50	50	50	99.9	0.7	636 (98.8%)
Dietary fiber (g)[d]	18	18	18	72	17	56 (8.7%)
Vitamin A (μgRAE)[c]	650	700	700	86	18	
Vitamin C (mg)[c]	100	100	100	91	16	396 (61.5%)
Vitamin D (mg)[e]	5.5	5.5	5.5	98	9	613 (95.2%)
Calcium (mg)[c]	650	650	650	85	17	261 (40.5%)
Iron (mg)[c]	10.5	10.5	6.5	85	16	254 (39.4%)
Potassium (mg)[d]	2600	2600	2600	94	10	396 (61.5%)
Magnesium (mg)[c]	270	290	290	90	11	235 (36.5%)
Disqualifying nutrients						
Added sugars (g)[f]	24.4	25.0	23.8	34	51	262 (40.7%)
Saturated fats (g)[dg]	15.2	15.6	14.8	18	21	189 (29.3%)
Sodium (g NaCl equivalent)[dh]	7	7	7	66	30	4 (0.6%)

NRF9.3, Nutrient-Rich Food Index 9.3; SD, standard deviation; RDVs, reference daily values; RAE, retinol activity equivalent.

[a] Values were derived from the Dietary Reference Intakes for Japanese, 2015 [1], except for added sugar, determined based on the World Health Organization's conditional recommendation [2].

[b] Estimated Energy Requirement for moderate level of physical activity.

[c] Recommended Dietary Allowance.

[d] Tentative dietary goal for preventing lifestyle-related disease.

[e] Adequate intake.

[f] The values were calculated by 5% of EER (World Health Organization's conditional recommendation).

[g] The values were calculated by 7% of EER (tentative dietary goal for preventing lifestyle-related disease).

[h] 7 g NaCl equivalent (g) = 2756 mg sodium.

References

[1] Ministry of Health, Labour and Welfare, Japan. Dietary Reference Intakes for Japanese, 2015. Available online: http://www.mhlw.go.jp/stf/seisakunitsuite/bunya/0000208970.html (accessed on Aug 15, 2021).

[2] World Health Organization. Guideline: Sugars intake for adults and children. Geneva: World Health Organization; 2015. Available online: http://apps.who.int/iris/bitstream/10665/149782/1/9789241549028_eng.pdf?ua=1 (accessed on Aug 15, 2021).
